# Correction for Dong et al., “Genomic revisitation and reclassification of the genus *Providencia*”

**DOI:** 10.1128/msphere.00288-24

**Published:** 2024-05-14

**Authors:** Xu Dong, Huiqiong Jia, Yuyun Yu, Yanghui Xiang, Ying Zhang

## AUTHOR CORRECTION

Volume 9, no. 3, e00731-23, 2024, https://doi.org/10.1128/msphere.00731-23. Page 4, Results, “Identification and characterization of novel species *Providencia zhijiangensis*,” last paragraph: “(N.L. masc. adj.)” should read “(N.L. fem. adj.)” as required by the grammatical rules for Latin species names.

Page 11: Following the guidelines of the International Code of Nomenclature of Prokaryotes (ICNP), a taxonomy section for *Providencia zhijiangensis* is required to comply with the necessary standards. Thus, the following taxonomic description, derived from the results, should be inserted at the end of Results.

### Description of *Providencia zhijiangensis* sp. nov

*Providencia zhijiangensis* (zhi.jiang.en’sis. N.L. fem. adj. *zhijiangensis*, pertaining to Zhijiang, a subdistrict within the Xihu District of Hangzhou City, Zhejiang Province, China, where the type strain was first isolated).

Cells are Gram stain-negative, motile, facultatively anaerobic rods. Colonies appear circular, raised, yellow, and opaque, with a smooth texture after 24 hours of incubation at 37°C on nutrient agar. Growth occurs between 15°C and 42°C, with optimal growth at 35°C and 37°C. Oxidase activity is absent. Produces acid from D-glucose, sucrose, and glycerol but not from D-mannitol, inositol, D-sorbitol, L-rhamnose, melibiose, amygdalin, D-arabinose, aesculin, arbutin, cellobiose, 2-ketogluconate, D-lyxose, salicin, or D-xylose. Positive for deaminase and negative for β-galactosidase, arginine dihydrolase, ornithine decarboxylase, lysine decarboxylase, and gelatinase. The Voges-Proskauer reaction and phenylalanine deaminase test are positive, while urease activity and indole production are negative. Citrate is utilized, but H_2_S is not produced. *Providencia zhijiangensis* can be distinguished from other *Providencia* species by its positive response to acetoin production tests and its lack of indole production. Notably, *P. zhijiangensis* does not utilize inositol and indole, which differentiates it from the clinically prevalent species *P. stuartii*, *P. rettgeri*, and *P. hangzhouensis*. The predominant fatty acids (>10%) are C16:0 and C14:0.

The type strain is D4759^T^ (= CCTCC AB 2023263^T^ = NBRC 116614^T^), isolated from a bile sample collected from a patient in the Zhijiang subdistrict of Hangzhou City, Zhejiang Province, China. The DNA G+C content is 42.91%.

