# Supplementary material for: Genomic revisitation and reclassification of the genus Providencia
Source: mSphere. 2024 Feb 27;9(3):e00731-23. doi: 10.1128/msphere.00731-23 (PMC10964429; doi:10.1128/msphere.00731-23)
Supplement: Supplemental figures and tables — Fig. S1-S12; Tables S1 and S2. [file msphere.00731-23-s0003.pdf]

Supplementary Materials for

Genomic revisitation and reclassification of the genus *Providencia*

Xu Dong *et al.*

Corresponding author: Ying Zhang, yzhang207@zju.edu.cn

The file includes:

Figure S1-S12

Table S1-S2

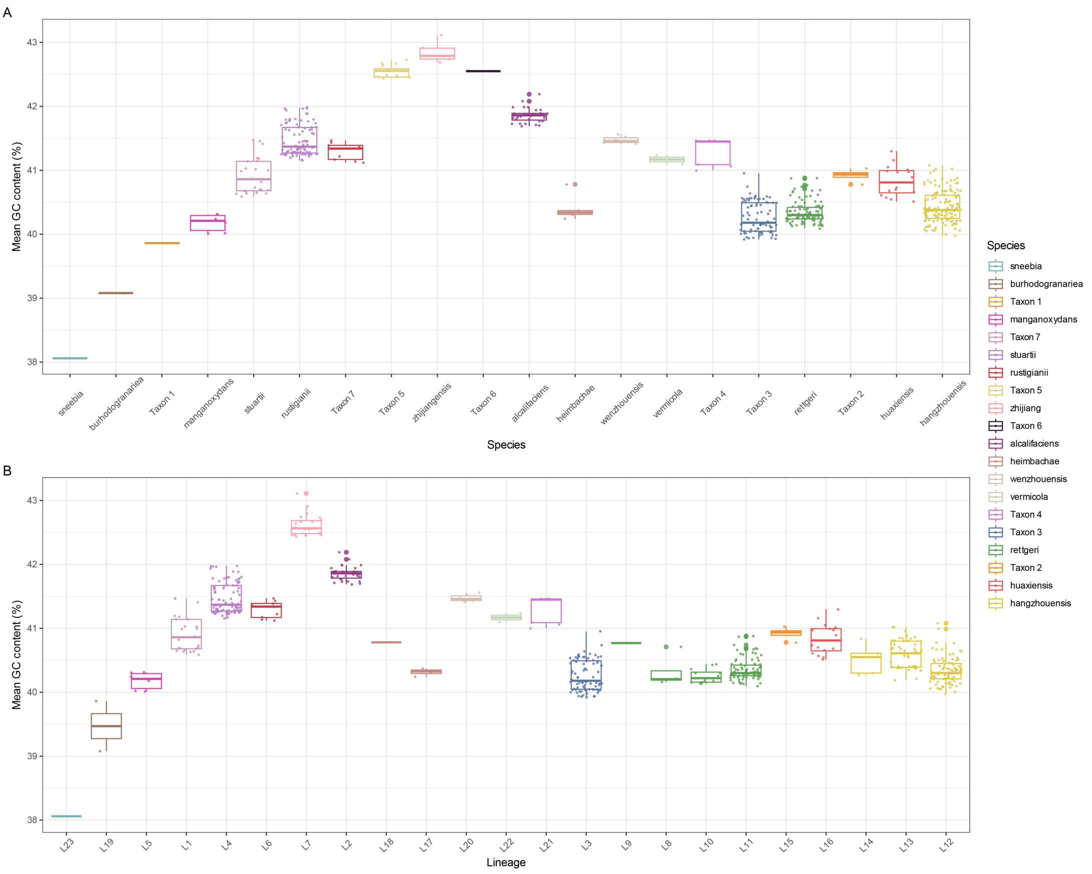

Figure S1. Box plot of GC content of each species **(A)** and each lineage **(B)**.

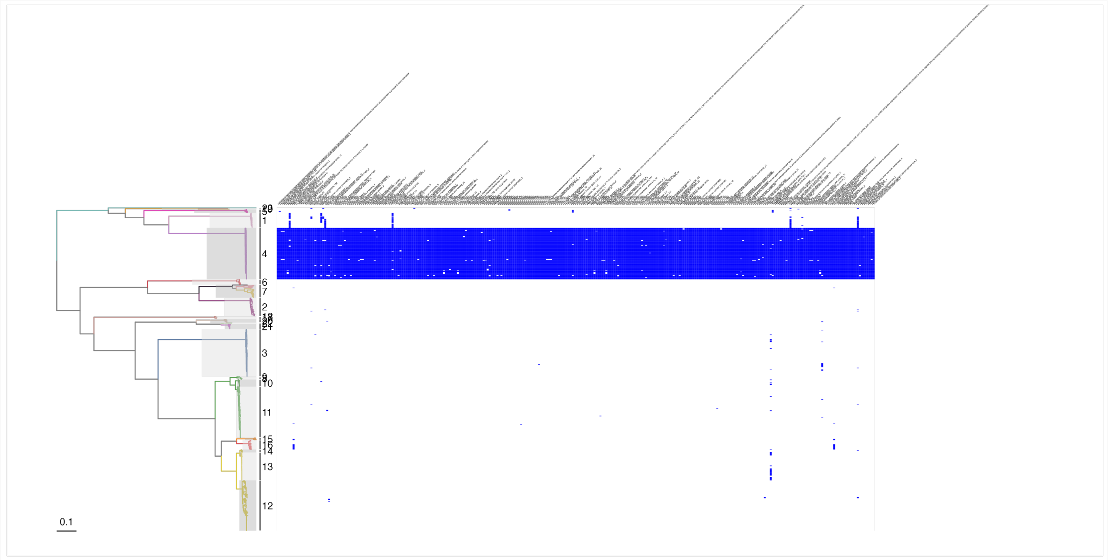

Figure S2. Intersection of core genes exclusive to *Providencia stuartii* (lineages 4), visualized in relation to the phylogenetic tree of the *Providencia* genus.

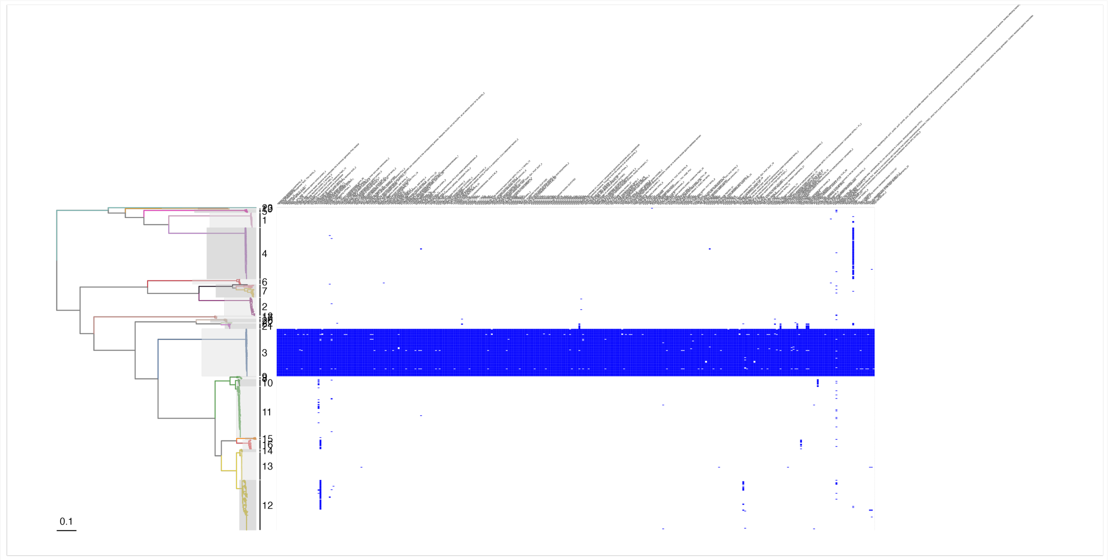

Figure S3. Intersection of core genes exclusive to Taxon 3 (lineages 3), visualized in relation to the phylogenetic tree of the *Providencia* genus.

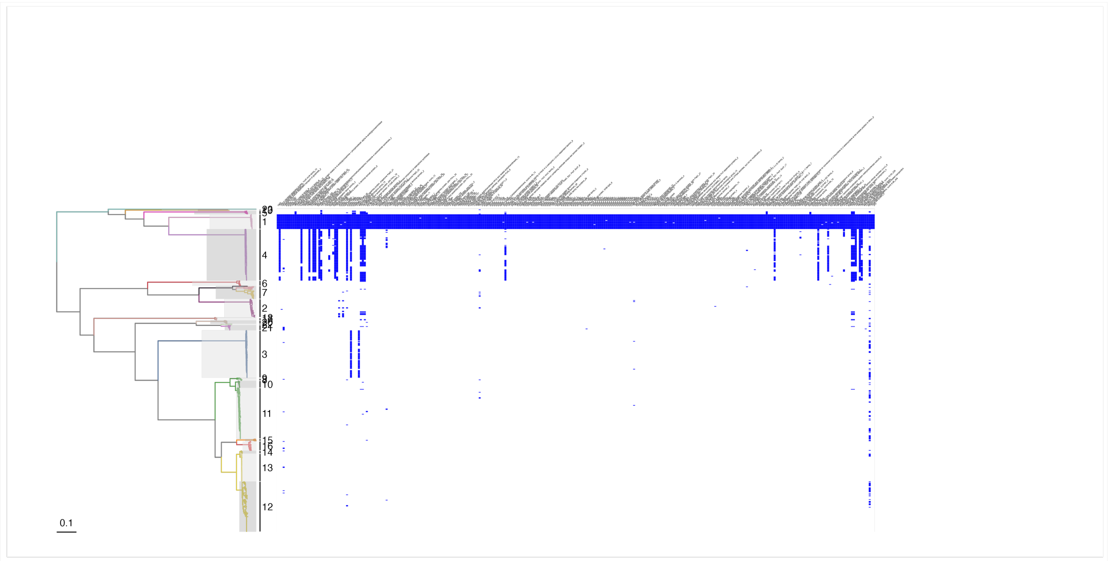

Figure S4. Intersection of core genes exclusive to Taxon 7 (lineages 1), visualized in relation to the phylogenetic tree of the *Providencia* genus.

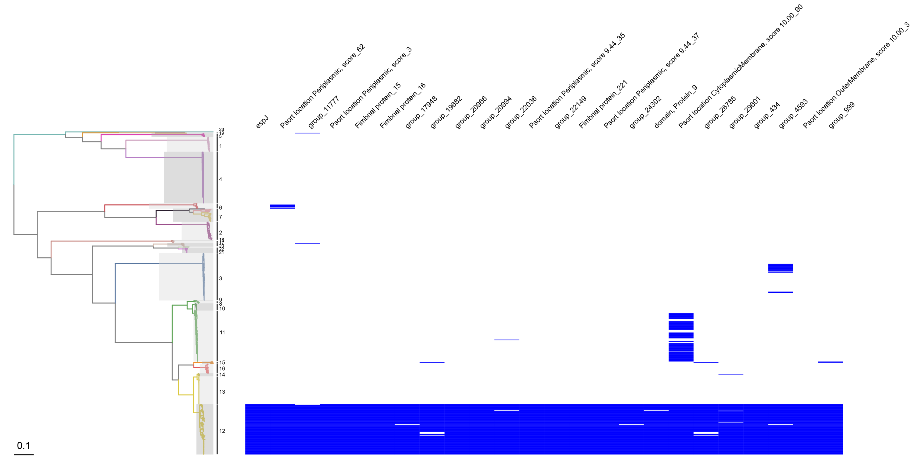

Figure S5. Intersection of core genes exclusive to *Providencia hangzhouensis* lineages 12, visualized in relation to the phylogenetic tree of the Providencia genus.

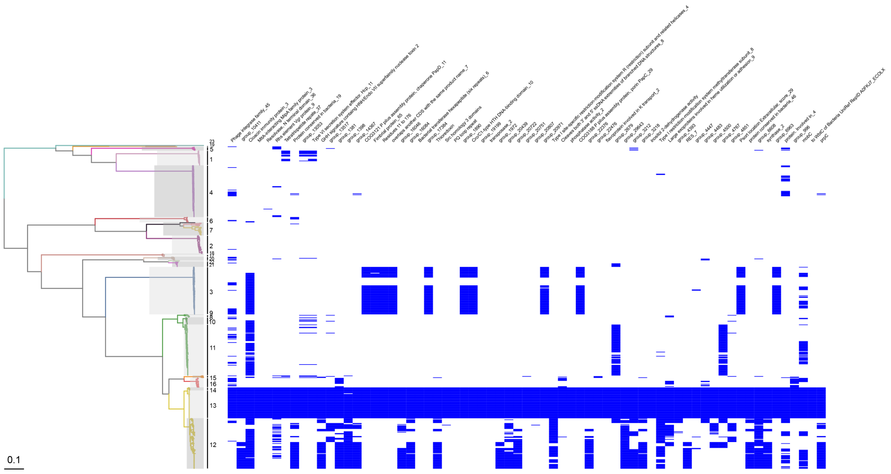

Figure S6. Intersection of core genes exclusive to *Providencia hangzhouensis* lineages 13-14, visualized in relation to the phylogenetic tree of the *Providencia* genus.

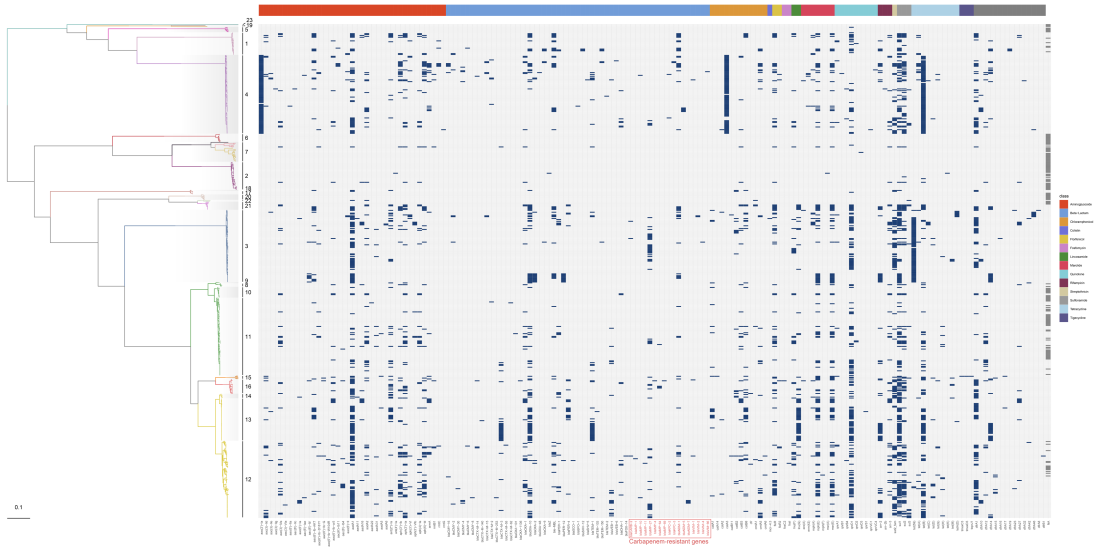

Figure S7. Distribution of antibiotic resistance genes in 545 strains. Different antibiotic profiles are indicated by different colors, where carbapenem resistance genes are marked by red boxes.

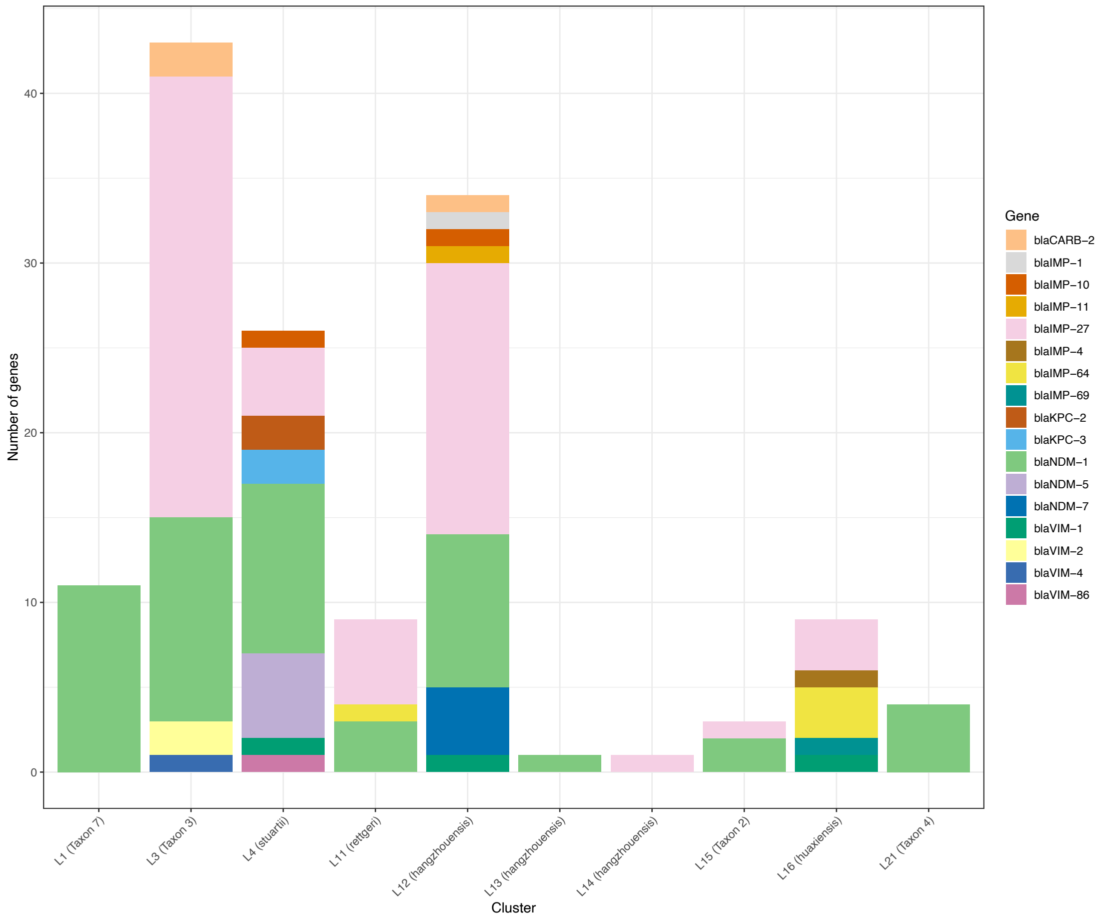

Figure S8. The number and types of genes carrying carbapenem-resistant genes in the lineage.

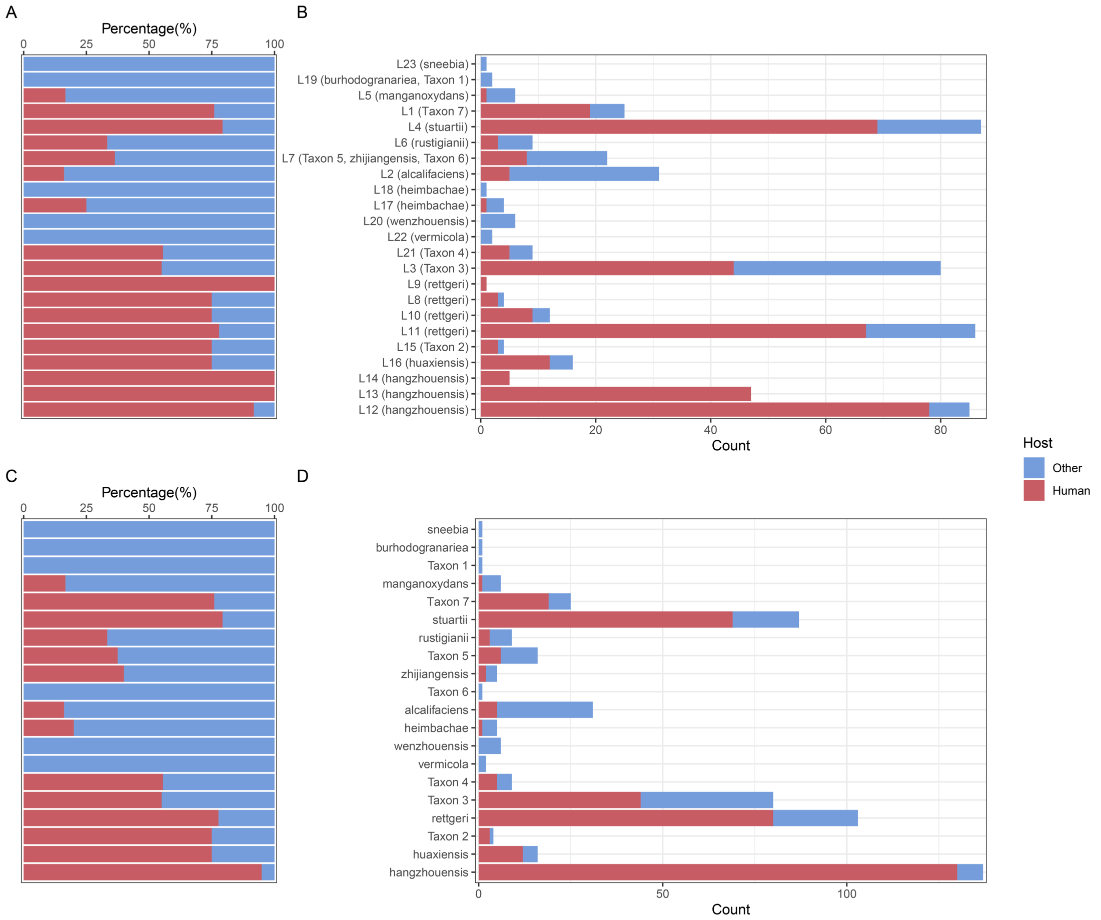

Figure S9. Number and percentage of human sources in each species/lineage. Panel **(A)** and **(C)** represent the percentage of human-orgin samples in each species and lineage, respectively. Panel **(B)** and **(D)** represent the number of human-orgin samples in each species and lineage, respectively. Red represents human sources, while other sources are classified as "other" and represented in blue.

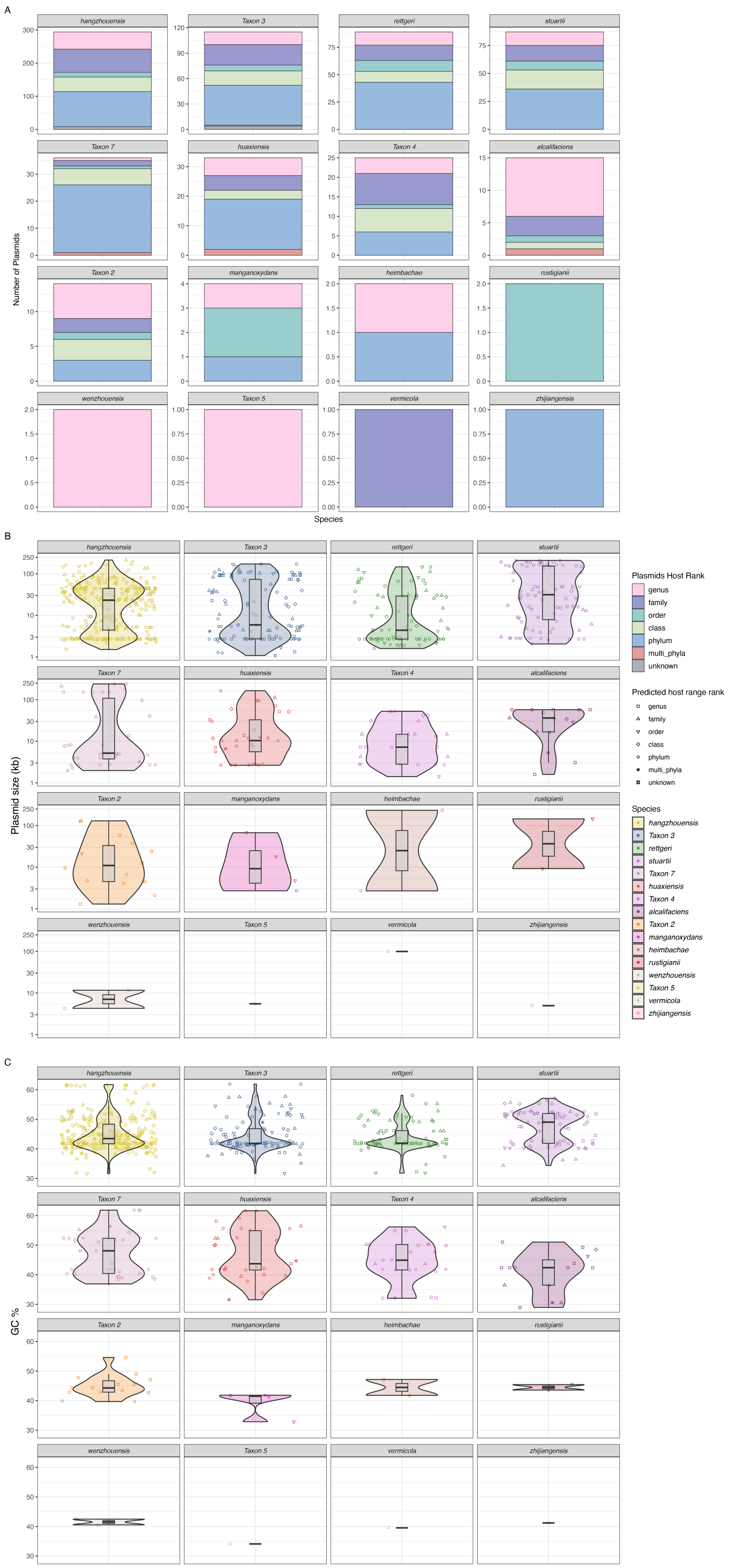
A
B
C
hangzhouensis
Taxon 3
rettgeri
stuartii
Taxon 7
huaxiensis
Taxon 4
alcalifaciens
Taxon 2
manganoxydans
heimbachae
rustigianii
wenzhouensis
Taxon 5
vermicola
zhijiangensis
Number of Plasmids
Species
Plasmid size (kb)
GC %
Plasmids Host Rank
genus
family
order
class
phylum
multi_phyla
unknown
Predicted host range rank
genus
family
order
class
phylum
multi_phyla
unknown
Species
hangzhouensis
Taxon 3
rettgeri
stuartii
Taxon 7
huaxiensis
Taxon 4
alcalifaciens
Taxon 2
manganoxydans
heimbachae
rustigianii
wenzhouensis
Taxon 5
vermicola
zhijiangensis

Figure S10. Characteristics of plasmids among species. (A) Predicted plasmid host. (B) Violin plot of plasmid size distribution. (C) Violin plot of plasmid GC content distribution.

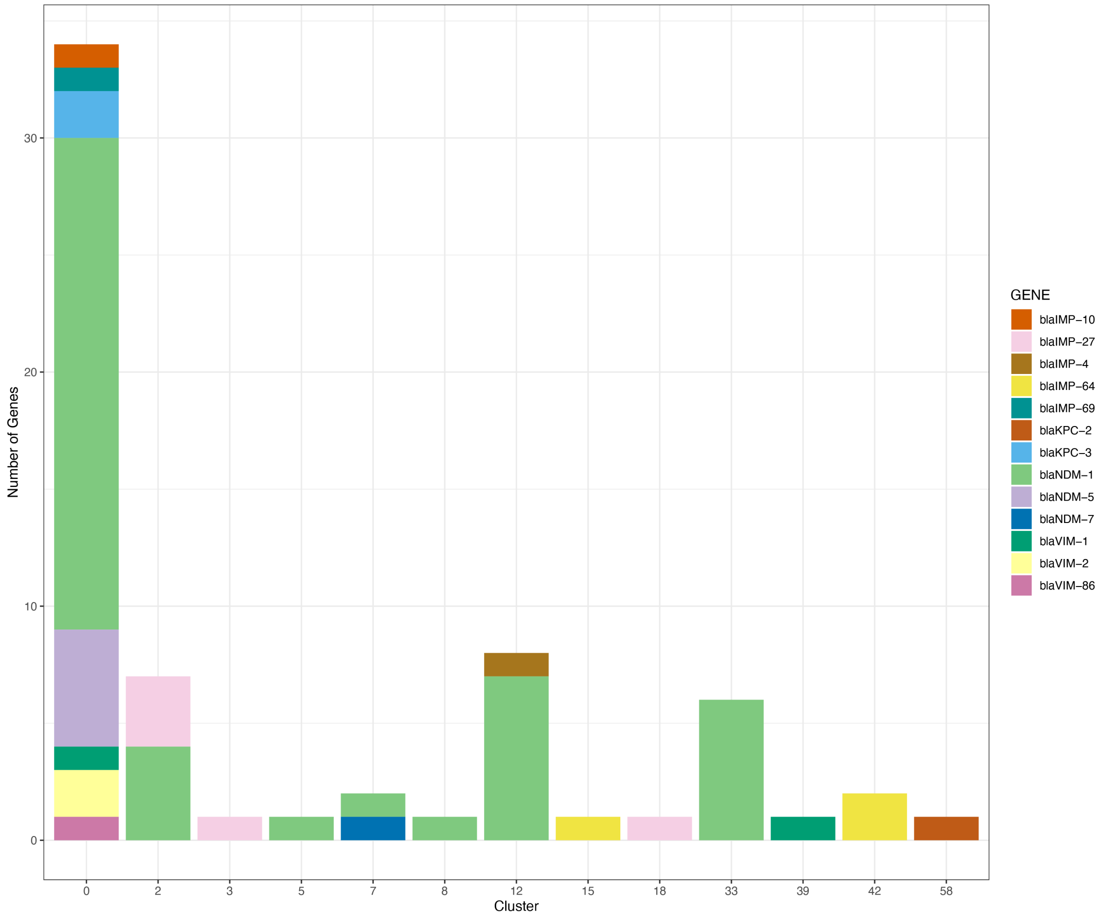

Figure S11. The number and types of genes carrying carbapenem-resistant genes in the different plasmid clusters.

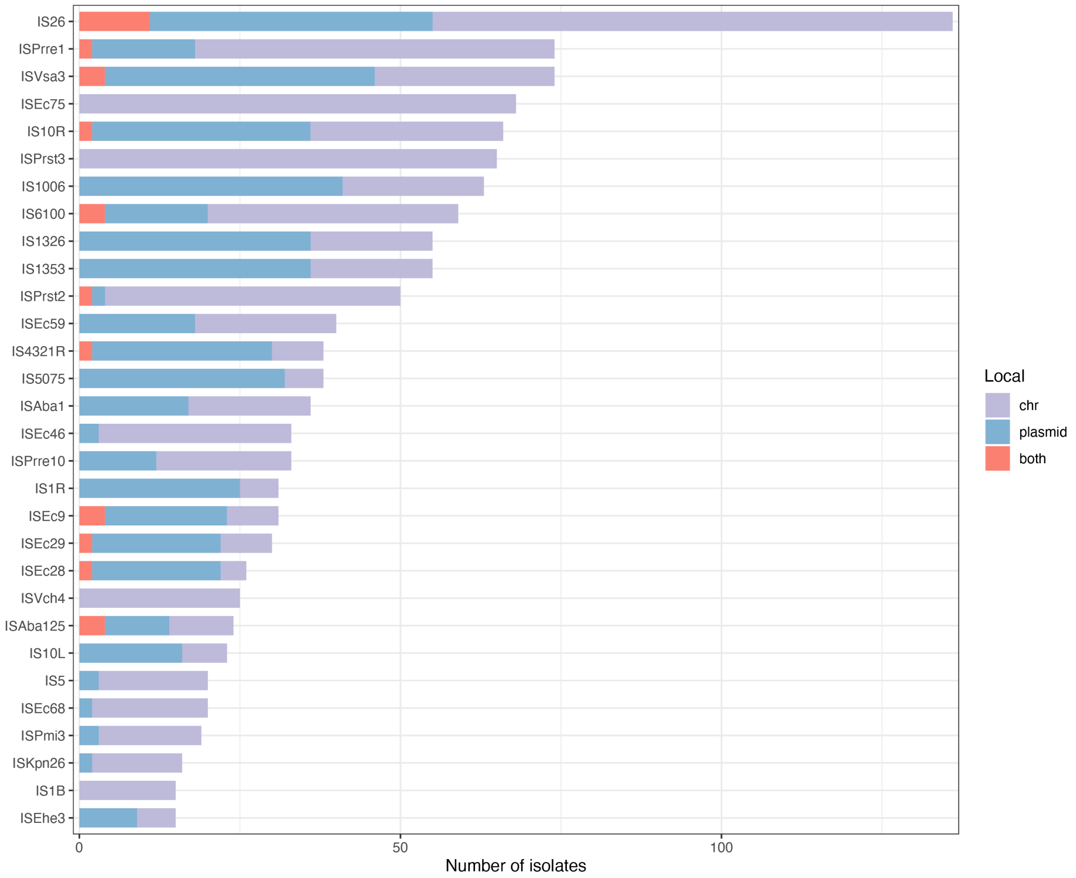

Figure S12. ISs and their locations across isolates of genera. ISs are ordered according to the number of isolates (top 30 greatest number).

Table S1 Antimicrobial susceptibility of strain D4759.

Antibiotics	MIC (mg/L)	Category[a]
Cefoxitin	4	S
Ceftazidime	0.06	S
Cefepime	<0.03	S
Cefuroxime	0.25	S
Gentamicin	0.5	S
Levofloxacin	1	S
Amikacin	2	S
Meropenem	0.06	S
Ampicillin	4	S
Aztreonam	<0.03	S
Imipenem	0.5	S
Kanamycin	2	S
Ciprofloxacin	0.25	S
Tigecycline	32	R

[a]: S, susceptible; R, resistant.

Table S2 Genomic characteristics of different *Providencia* species.

phylo group	Taxonomic assignment	Genome Size (Mb)[a]	G + C mol%	No. of CDS[b]	No. of genomes
1	*Providencia stuartii*	4.42	41.47	4067	87
2	**Taxon 7**	4.32	40.92	3853	25
3	*Providencia manganoxydans*	4.49	40.12	4163	6
4	*Providencia burhodogranariea*	4.58	39.08	3892	1
5	**Taxon 1**	4.86	39.86	4585	1
6	*Providencia sneebia*	3.90	38.06	3230	1
7	*Providencia hangzhouensis*	4.61	40.43	4219	137
8	***Providencia zhijiangensis***	3.85	42.85	3487	5
9	**Taxon 2**	4.57	40.92	4158	4
10	*Providencia rettgeri*	4.44	40.35	4041	103
11	**Taxon 3**	4.65	40.27	4244	80
12	*Providencia huaxiensis*	4.53	40.83	4151	16
13	*Providencia wenzhouensis*[d]	4.72	41.47	4351	6
14	**Taxon 4**	4.72	41.32	4362	9
15	*Providencia alcalifaciens*	4.11	41.86	3796	31
16	*Providencia heimbachae*	4.29	40.41	3912	5
17	*Providencia vermicola*	4.43	41.17	3972	2
18	*Providencia rustigianii*	3.87	41.29	3557	9
19	**Taxon 5**	4.01	42.55	3675	16
20	**Taxon 6**	3.93	42.55	3502	1

a: Average genome size within species

b: Average coding proteins (CDS) number within species
