# Supplementary material for: Genomic revisitation and reclassification of the genus Providencia
Source: mSphere. 2024 Feb 27;9(3):e00731-23. doi: 10.1128/msphere.00731-23 (PMC10964429; doi:10.1128/msphere.00731-23)
Supplement: Descriptions — of Data S1 and S2. [file msphere.00731-23-s0004.pdf]

Description of Additional Supplementary Files

File Name: Supplementary Data 1

Description: Metadata for the isolates included in this study. The 'Species' column represents the accurate species classification post-revision, while the 'Labelled organism_name' column indicates the original designation in NCBI. Isolates with classification discrepancies are highlighted in yellow.

File Name: Supplementary Data 2

Description: Details of plasmid sequences analyzed in this study.
